# Can Digital Financial Inclusion Promote Green Innovation in Heavily Polluting Companies?

**DOI:** 10.3390/ijerph19127323

**Published:** 2022-06-15

**Authors:** Long Xue, Xuemang Zhang

**Affiliations:** School of Economics and Management, Zhengzhou University of Light Industry, Science Avenue 136, Zhengzhou 450000, China; xuemang_z@163.com

**Keywords:** digital financial inclusion, green innovation, financing constraints, life cycle

## Abstract

This paper takes the China A-shares listed companies in heavy polluting industries from 2011 to 2020 as samples, combines the digital financial inclusion index to empirically examine the impacts of digital financial inclusion development on the green technology innovation of heavily polluting companies, and reveals its mechanism of action and its heterogeneity of the impacts of enterprises’ green technology innovation in different development stages. The empirical research results show that the development of digital financial inclusion is able to promote the green innovation of heavy-polluting enterprises. Its main manifestation is that the development of digital financial inclusion helps the increase of green patent applications of heavy-polluting enterprises. This conclusion is validated through the endogeneity and robustness tests. The test results of the mechanism of action show that digital financial inclusion promotes green innovation of enterprises by alleviating corporate financing constraints and financial mismatch problems. Further research results show that the role of digital financial inclusion in promoting green technology innovation in heavy-polluting enterprises is more pronounced in mature enterprises. Therefore, this study provides a theoretical basis for the development of digital financial inclusion to promote heavy-polluting enterprises to achieve green transition through green technology innovation, thus achieving the “dual carbon” goal.

## 1. Introduction

Since the Chinese government proposed the “dual carbon” goal at the 75th United Nations General Assembly in 2020, how to achieve this goal has become a hot issue for policy-makers and academia [1,2]. High-speed economic development brought about by heavy-polluting enterprises at the expense of the environment pollution will bring serious environmental issues [3]. Therefore, whether they can achieve green transition is critical to achieving the “dual carbon” goal on schedule. Green technology innovation is a general term for new technologies, new processes, and new products that help reduce environmental pollution and save resources. Therefore, it is one of the key paths for heavy-polluting enterprises to achieve green transition. However, green technology innovation requires stable and continuous funds. There is a high degree of uncertainty, which leads to the problem of strong financing constraints for heavy-polluting enterprises to implement green technology innovation. Digital financial inclusion is an action to promote financial inclusion through the use of digital financial services [4]. In recent years, digital inclusive finance has developed rapidly, driven by modern information technologies, such as mobile payment, big data, and cloud computing. Digital inclusive finance has broken the boundaries of traditional financial services and alleviated information asymmetry in traditional financial services. The resulting high cost has broken through the limitations of time and geography and further expanded the reach and service depth of inclusive finance. Therefore, the development of digital inclusive finance provides the possibility of financial support for heavily polluting enterprises to implement green technology innovation. In the existing literature, although some scholars have paid attention to the influencing factors of corporate green technology innovation and the impact of digital financial inclusion on it [5,6,7,8], few scholars have studied whether digital financial inclusion can promote green technology innovation in heavy-polluting enterprises, and no scholar has revealed its mechanism of action and its heterogeneity. It can be seen from the above sentences that the main contributions of this paper are: First, taking heavy-polluting enterprises as the research object, it reveals the influencing mechanism of digital financial inclusion on the green innovation of heavy-polluting enterprises from the perspectives of financing constraints and financial mismatches, which not only expands the relevant literature on the impact of digital financial inclusion on enterprises, but also provides a new perspective for analyzing the influencing factors of enterprise green innovation. Second, on the basis of systematically expounding how digital financial inclusion affects the green innovation mechanism of enterprises, it further explores whether this impact will be affected by the heterogeneity of the enterprise life cycle, and clarifies the differences in the effects of digital financial inclusion on green innovation for enterprises at different development stages. This research has important theoretical and practical significance for government supports of the development of digital financial inclusion and promotion of the green technology innovation in heavy-polluting enterprises, and in turn it can promote the green transition of heavy-polluting enterprises, and help China achieve the “dual carbon” goal.

## 2. Literature Review and Hypothesis

### 2.1. Green Innovation

In the context of the “two-carbon” goal, green innovation, as an important activity to promote sustainable environmental development, has received extensive attention from scholars. A review of the existing research finds that the factors affecting the green innovation of enterprises are mainly concentrated in two aspects: environmental effects and resource supply. In terms of environmental effects, Lyubich et al. believe that when companies face higher environmental standards, they will be more inclined to develop and apply clean technologies to reduce the environmental pollution caused by their productions and operations and implement environmental regulations such as environmental taxes [9]. It is an effective way to improve environmental standards. Yu Lianchao et al. found that environmental taxes can effectively promote the green innovation of enterprises [10], which verifies the views of Lyubich et al. Furthermore, environmental regulations represented by taxes stimulate the green innovation of enterprises, and the incentive effect varies by differences in types of firms and regions [11]. The above research shows that scientific and reasonable environmental regulations and the policy mix can actively guide and motivate enterprises to carry out green technology innovation [12]. In addition to the abovementioned mandatory environmental effects, incentive-based environmental measures also have a positive impact on green innovation [11], such as government subsidies and some voluntary agreements [13,14]. In terms of resource supply, green innovation is characterized by high investments, long cycles, and high benefit uncertainty compared with other innovation activities and requires greater resource investment [15]. Green innovation resources, such as FDI, environmental investment, and foreign investment, have a positive impact on green innovation behavior and efficiency [16,17], and if the green loan interest rate provided by banks is lower than the threshold, enterprises tend to accept bank loans to implement green innovation [18]. In addition, corporate financial and governance status is also a cornerstone of green innovation resources. Good finance can provide sufficient material and human resources for green innovation [19]. The environmental protection orientation of corporate stakeholders and equity financing are also conducive to green innovation [20,21].

### 2.2. Digital Financial Inclusion

As an important engine for the country’s high-quality development, digital inclusive finance provides new impetus and opportunities for promoting economic development [22], and its stable development has important macro and micro impacts. At the macro level, the development of digital inclusive finance has positive significance for promoting entrepreneurship, stimulating residents’ consumption, accelerating inclusive growth, narrowing the urban-rural income gap, and driving the digital innovation of commercial banks [23,24,25,26,27]. At the microlevel, digital inclusive finance can, on the one hand, carry out an accurate risk assessment for enterprises under conditions of digital technology [28] and, on the other hand, enhance the information processing capabilities of financial institutions and investors, effectively reducing the relationship between enterprises and the market. The problem of information asymmetry between enterprises [29] increases the source channels and availability of funds for enterprises, thereby easing their financing constraints. In addition, some scholars have paid attention to the significant impact of digital financial inclusion on corporate innovation. The development of digital inclusive finance can alleviate the financing constraints and financial mismatches faced by enterprises and promote enterprise innovation through R&D investment, the accumulation of redundant expansion effects, and human capital upgrade effects [29,30,31].

### 2.3. Digital Financial Inclusion and Green Innovation

A large number of studies have shown that digital financial inclusion has a positive impact on corporate innovation activities, investment, and income [29,32]. Specifically, from the perspective of green innovation, the development of digital finance promotes urban economic agglomeration and optimizes the regional financial structure, provides a good external financial environment for the development of enterprises’ green innovations, eases the financing constraints faced by enterprises [33], and then promotes enterprises’ green innovations [34]. Jiang Jianxun et al. proved this conclusion from the perspective of new energy enterprises [35]. Based on the above research, we believe that the development of digital financial inclusion has a similar positive effect on the green innovation of heavy-polluting enterprises.

The Porter hypothesis proposes that innovation can offset the cost of complying with environmental requirements, and when the environmental system can be improved, it can further guide enterprises toward innovation. The mainstream view holds that both mandatory and incentive environmental effects can have a positive impact on corporate green innovation [10,14]. Currently, however, for heavily polluting enterprises mainly concentrated in the manufacturing sector, green innovation is closely related to capital market opening [36], resource acquisition and input [16], and the Green Credit System is formulated for the sustainable development of the environment and the transformation of green development of enterprises. The implementation of green credit policies, such as the “Guidelines”, has changed the external credit environment, making it more difficult to obtain loans and increasing the financing constraints of such enterprises [37]. In addition, there is currently no complete green innovation mechanism in China to ensure that enterprises obtain public resources. Due to the nature of the industry, it is relatively difficult for heavily polluting enterprises to obtain public resources, such as government subsidies, which also deepens the financing constraints and weakens the incentive to engage in green innovation and, thus, is not conducive to the green innovation output of heavily polluting enterprises [38]. According to the theory of resource allocation efficiency, however, the limited financial capital in the market should flow into high-efficiency enterprises to achieve the overall effective allocation of resources and Pareto optimality, the misallocation of financial resources is a deviation from “effective allocation” [30]. The external financing that heavily polluting enterprises can obtain is limited. The main financing methods come from bond financing provided by financial institutions such as banks and equity financing in the capital market [39]. This type of financing not only pays more attention to the ability of enterprises to obtain profits and competitive advantages but also considers whether the funds can be recovered safely and whether the loan income is higher than the cost, including time value, etc., and is also affected by policy bias. Economic development allocates more financial resources to projects with quick returns and short cycles, while green innovation and development are characterized by long cycles, high costs, unclear market demands, and uncertain returns, making them vulnerable to misallocation of financial resources [30].

The financial environment is one of the main factors affecting the business activities of enterprises, and the supply of financial resources significantly affects the progress of business activities [40]. Compared with the “backward-looking” preference of the traditional credit model, the development of digital inclusive finance has effectively corrected the “stage mismatch” in the traditional financial system, making it easier for enterprises to obtain financial resources than before; that is, the development of digital inclusive finance can hedge financing. The “financial dilemma” was brought about by constraints [41]. First, the issue of information asymmetry is a leading problem that results in corporate financing constraints. Digital financial inclusion can reduce the information asymmetry between investors and companies by virtue of its digital technology [42]. Furthermore, digital inclusive finance makes up for the under covered long-tail groups in the traditional financial model and incorporates small and medium-sized enterprises, individual industrial and commercial households, and individual investors who are not able to participate in investment under the traditional financial model into the new financial system. It increases the investor group and broadens the source of funds, which effectively alleviates the problem of corporate financing constraints [29]. Second, digital inclusive finance improves the ability of information collection and integration. Through the capture and analysis of corporate behavioral data, it is possible to conduct more accurate risk assessments for enterprises and then strengthen the control of risks and credit, thus, forcing traditional transformation and upgrading of financial institutions to alleviate the misallocation of financial resources and improve the supply efficiency of financial resources [30]. Third, digital inclusive finance relies on its artificial intelligence technology, big data technology, machine learning technology, and other emerging technologies to make the production and operation of heavily polluting enterprises more scientific and efficient. It also contributes to the organizational structure becoming more rational and flexible, thereby reducing the cost of enterprises and expenses to provide more capital space for green innovation and ensure the smooth progress of green innovation of enterprises [31]. Based on the above analysis, the following hypothesis is proposed:

**Hypothesis** **1a** **(H1a).** *Digital financial inclusion effectively promotes green innovation in enterprises.*

**Hypothesis** **1b** **(H1b).** *Digital inclusive finance promotes the green innovation of enterprises by reducing the financing constraints and financial mismatches faced by enterprises.*

## 3. Variables, Data and Methods

### 3.1. Variable Setting

#### 3.1.1. Explanatory Variables

Digital financial inclusion (Dindex). This paper uses the China Digital Financial Inclusion Index released by the Digital Finance Research Center of Peking University to measure the development degree of digital financial inclusion [43]. In addition, in order to ensure the accuracy of the research, we matched the prefecture-level city digital financial inclusion index with the enterprise micro-data (the selection of cities is based on those enterprises’ registration places).

#### 3.1.2. Dependent Variable

Green innovation (Patent). Due to the special nature of the industry, heavily polluting enterprises will inevitably cause some damage to the ecological environment. Their innovative output to eliminate or reduce pollution and damage caused to the ecological environment and weaken negative ecological effects is called green innovation. Referring to the practice of Wang Xin and Wang Ying [44], this paper adopts the natural logarithm method to measure the green innovation of enterprises by adding 1 to the number of green patent applications in the current year.

#### 3.1.3. Mechanism Variables

(1) Financing constraints (Sa). In the current research on financing constraints in academia, KZ, WW, and Sa indices all measure the degree of financing constraints, but forSa, the basic data used in the calculation of the index are the scale and age of the enterprise, which has a strong exogenous and objective nature. Therefore, this paper refers to the research of Hadlock and Pierce [45], using an Sa index to measure the financing constraints faced by firms. The index calculation method is $\mathrm{Sa}=-0.737\times \mathrm{Size}+0.043\times {\mathrm{Size}}^{2}-0.040\times \mathrm{Age}$. (2) Financial mismatch (Fm). Limited financial capital in the market should flow into high-efficiency enterprises to achieve the effective allocation of overall resources, while the misallocation of financial resources is a deviation from the “effective allocation”. This paper draws on the research of Shao Ting [46], using the cost of capital of enterprises. The financial mismatch is measured by the degree of deviation from the industry average cost of capital. The calculation method of this indicator is [Interestexpense/(Debt−Accountspayable)]/Industryaveragecostofcapital.

#### 3.1.4. Control Variables

This paper controls the variables at the financial and governance levels that may affect the green innovation of enterprises. The control variables at the financial level include enterprise scale (Size), which is the natural logarithm of the total assets of the enterprise; asset-liability ratio (Lev), which is the ratio of total liabilities to total assets; return on assets (Roa), which is the ratio of net profit to total assets; and fixed asset ratio (PPe), which is the ratio of net fixed assets to total assets. The control variables at the governance level include the combination of two positions (Dual); if the chairman and the general manager are the same person, the assignment is 1, otherwise, the assignment is 2; management shareholding (Msh) is the ratio of the number of shares held by management to the total number of shares; shareholding concentration ratio (Top) is the sum of the shareholding ratios of the top ten shareholders of the enterprise; and shareholding balance ratio (Balance) is the ratio of the sum of the shareholding ratios of the second-largest shareholder to the tenth largest shareholder and the shareholding ratio of the first largest shareholder. In addition, this paper also controls the industry effect (Ind) and year effect (Year) by setting dummy variables.

### 3.2. Data

This paper takes the China A-shares listed companies in heavy polluting industries from 2011 to 2020 as samples. The Digital Financial Inclusion Index, which is derived from the “Peking University Digital Financial Inclusion Index,” compiled and released by the Peking University Financial Research Center. The green patent data used to measure green innovation is from the China Research Data Service Platform (CNRDS). The Guotai Security Database (CSMAR) provided the data on governance and financial aspects of heavy polluting industries. In addition, considering the quality of observation samples, this paper adopts the following principles to screen and process the data of listed companies: (1) The sample data of ST and ST* companies are excluded; (2) the sample data of the year with missing data were excluded to ensure the comparability of the data; (3) to eliminate the influence of outliers, this paper performs the Winsorization process on the 1% quantile of the data at the enterprise level.

### 3.3. Methods

#### 3.3.1. Full Sample Regression

In order to explore how digital financial inclusion affects the green innovation of enterprises, this paper refers to the practice of Wan Jiayu et al. to construct a two-way fixed effect Model (1), and conducts an estimation test on the sample data [29].

(1) $${\mathrm{Patent}}_{\mathrm{it}}=\alpha_{0}+\alpha_{1}\times {\mathrm{Dindex}}_{\mathrm{it}}+\alpha_{2}\times {\mathrm{Controls}}_{\mathrm{it}}+\Sigma \mathrm{Year}+\Sigma \mathrm{Ind}+\varepsilon_{\mathrm{it}}$$

Model (1) Patent represents the green innovation of heavily polluting enterprises; $\alpha_{0}$ represents the constant term of the equation; Dindex represents digital financial inclusion; Controls represents a set of corporate governance and financial control variables selected in this paper; Year and Ind represent the year dummy variable and industry dummy variable, respectively, used to control for time effects and industry effects; and ε represents a random error term. In Model (1), we focus on the preregression coefficient $\alpha_{1}$ of digital financial inclusion. If the coefficient is significantly greater than zero, it indicates that digital financial inclusion will promote green innovation in heavily polluting enterprises; thus, H1a of this paper is verified.

#### 3.3.2. Mechanism Effect Regression

In order to verify that financing constraints and financial mismatches are the mechanisms by which digital financial inclusion affects green innovation, this paper introduces a cross multiplication Model (2) of digital financial inclusion, financing constraints, and financial mismatches on the basis of Model (1).

(2) $$\begin{array}{cc} {\mathrm{Patent}}_{\mathrm{it}} & =\lambda_{0}+\lambda_{1}\times {\mathrm{Dindex}}_{\mathrm{it}}\times \mathrm{Sa}/{\mathrm{Fm}}_{\mathrm{it}}+\lambda_{2}\times {\mathrm{Dindex}}_{\mathrm{it}}+\lambda_{3}\times \mathrm{Sa}/{\mathrm{Fm}}_{\mathrm{it}}\\ & +\;\lambda_{4}\times {\mathrm{Controls}}_{\mathrm{it}}+\Sigma \mathrm{Year}+\Sigma \mathrm{Ind}+\varepsilon_{\mathrm{it}} \end{array}$$

Consistent with the meaning of Model (1), except for digital financial inclusion and financing constraints, the multiplication term of financial mismatch (Dindex × Sa/Fm) and mechanism variable financing constraints (Sa) and financial mismatch (Fm) are added. In Model (2), we mainly focus on the regression coefficient before the multiplication term $\lambda_{1}$. If the coefficient is significantly greater than zero, it indicates that the interactive effect of digital financial inclusion, financing constraints, and financial mismatch promotes green innovation; that is, the mechanism by which digital inclusive finance affects green innovation is through financing constraints and financial mismatch.

## 4. Analysis of Empirical Results

### 4.1. Descriptive Statistical Characteristics

Table 1 reports the descriptive statistics of the main variables. From this, it can be found that the maximum value of green innovation in the selected sample is 3.970, far exceeding the average value of 0.736. However, there are also samples with green innovation values of zero, which shows that there are large differences in the degree of green innovation between different enterprises. The standard deviation of digital inclusive finance is 0.718, indicating that digital financial inclusion is relatively concentrated near the average value, but the difference between the maximum value and the minimum value is still large. Judging from the descriptive statistical results of enterprise-level data, because the selected sample is a heavily polluting industry concentrated in manufacturing enterprises, its asset-liability ratio and fixed assets ratio are 0.426 and 0.311, respectively, which are relatively higher than those of other industries.

### 4.2. Collinearity and Correlation Test

To avoid the error caused by multicollinearity in the regression results, this paper conducts a VIF test among the variables. The test results are shown in the first column of Table 2, and the VIF values are all less than 10. Table 2 lists the correlation analysis results. It can be seen from the table that the correlation coefficient between digital financial inclusion (Dindex) and green innovation (Patent) is 0.181, which is significant at the 1% level, providing preliminary validation of this paper. The main assumption is that digital financial inclusion can effectively promote green innovation in heavily polluting enterprises. On the whole, the highest correlation coefficient between digital financial inclusion and each control variable is 0.127, and the absolute value of the correlation coefficient between each control variable does not exceed 0.5, which is a low level. The above results show that the control variables selected in this paper do not have serious multicollinearity problems, and the regression results obtained are robust and reliable.

### 4.3. Benchmark Regression

Table 3 reports the regression results of digital financial inclusion (Dindex) and green innovation (Patent) in Model (1), where column (1) is the regression result without control variables and column (2) is the regression result with control variables. The results show that the coefficients of digital financial inclusion are 0.2882 and 0.0031, respectively, both of which are significant at the 1% level, indicating that the development of digital financial inclusion can promote green innovation in heavily polluting enterprises.

Considering the endogeneity problem, this paper uses the independent variable with one lag period (Tool) as an instrumental variable to re-estimate the model by the two-stage least squares method. Column (3) is the regression result of the first stage after adding instrumental variables, tool variables and digital financial inclusion are significantly correlated at the 1% level, which fulfills the requirement of correlation of instrumental variables. Column (4) is the regression result of the second stage. Digital financial inclusion has a positive impact on the green innovation of enterprises at the 1% level. In summary, in the benchmark regression and the two-stage least squares regression, the coefficient of digital financial inclusion is significantly positive, indicating that the development of digital financial inclusion can effectively promote the green innovation of heavily polluting enterprises.

### 4.4. Robustness Test

To ensure the reliability of the previous conclusions, the aspects that may have an impact are considered here, namely, the variable measurement problem and the dynamic problem, and the solutions are as follows. (1) Replacing the explained variables, innovation is divided into innovation input and innovation output. As a necessary condition for innovation output, innovation input can also reflect the green innovation of enterprises. Therefore, the green innovation of enterprises is remeasured from the perspective of innovation input. Drawing on the practice of Guo Ping [47], the proportion of R&D investment in the company’s main business income (RD) is used as a proxy variable for corporate green innovation (Patent), and the regression results are shown in column (1) in Table 4. There was a significant positive correlation between the main variables. (2) Replacing explanatory variables, the prefecture-level city digital financial inclusion index is used to measure the explanatory variables in the previous article. Considering the problem of regional development, the provincial digital financial inclusion index (PDindex) is used here as a proxy for digital financial inclusion (Dindex) variables, the regression results are shown in column (2) in Table 4, and there is a significant positive correlation between the main variables. (3) For the explanatory variables with one lag period, considering the endogeneity caused by the dynamic problem, the explanatory variables with one lag period are brought back into the model for regression, and the regression results are shown in column (3) of Table 5. There was a significant positive correlation between them. In summary, the conclusions of this paper are robust.

### 4.5. Examination of the Mechanism of Action

Combined with the previous theoretical analysis, this paper believes that digital financial inclusion may promote green innovation in heavily polluting enterprises by alleviating the financing constraints at the enterprise level and financial mismatch at the market level. Therefore, this paper examines the impact mechanism of digital financial inclusion on the green innovation of heavily polluting enterprises from the perspectives of financing constraints and financial mismatch.

The impact of the interaction between digital financial inclusion and financing constraints (Dindex×Sa) and financial mismatch (Dindex×Fm) on green innovation is shown. The regression results show that the regression coefficient before the multiplication of digital financial inclusion and financing constraints is 0.1307, which is significant at the 10% level, and the regression coefficient before the multiplication of digital financial inclusion and financial mismatch is 0.0650, also at the 10% level. The above results show that financing constraints and financial mismatches are the mechanisms by which digital inclusive finance affects green innovation. In addition, this paper tests the action mechanism of the subdivision index coverage (Dcover) of digital financial inclusion to further verify the effectiveness of the action mechanism of financing constraints and financial mismatch. The test principle is the same as the above, and the results are shown in columns (3) and (4) of Table 5. It can be seen that the regression coefficients before the multiplication of digital inclusive finance coverage and financing constraints (Dcover×Sa) and financial mismatch (Dcover×Fm) are 0.1508 and 0.0601, respectively, both of which are significant at the 10% level. The above results show that digital inclusive finance alleviates financing constraints and financial mismatches through coverage, thereby promoting green innovation and verifying H1b.

**Table 5:** Mechanism of action test.

Variable	(1)	(2)	Variable	(3)	(4)
	Dindex			Dcover	
	Patent	Patent		Patent	Patent
Dindex × Sa	0.1307 *		Dcover × Sa	0.1508 *	
	(1.65)			(1.84)	
Sa	0.6681 ***		Sa	0.6672 ***	
	(10.65)			(10.62)	
Dindex×Fm		0.0650 *	Dcover×Fm		0.0601 *
		(1.83)			(1.65)
Fm		−0.0472 *	Fm		−0.0477 *
		(−1.65)			(−1.67)
Dindex	0.3185 ***	0.3845 ***	Dcover	0.2282 ***	0.2791 ***
	(5.38)	(6.19)		(5.07)	(5.93)
Controls	YES	YES	Controls	YES	YES
Cons	−5.8539 ***	−9.2352 ***	Cons	−5.7958 ***	−9.1828 ***
	(−14.98)	(−32.47)		(−14.85)	(−32.29)
Year	YES	YES	YES	YES	YES
Ind	YES	YES	YES	YES	YES
N	4799	4799	N	4799	4799
Adj-R^2^	0.3441	0.3377	Adj-R^2^	0.3437	0.3370

Note: Robust standard errors are in parentheses. *, **, and *** indicate significance at the 10%, 5%, and 1% levels, respectively.

### 4.6. Further Research

Based on the above analysis, we believe that digital financial inclusion has a greater effect on promoting green innovation in more mature enterprises. Enterprises with different life cycles have different characteristics, with obvious differences in market pressure, capital demand, financing constraints, and risk management [48]. Relatively speaking, enterprises in the growth stage have good development prospects, high growth potential, and high investment returns. They are expected to become benchmark enterprises in the industry in the future, and it is the best time for enterprises to realize the expansion of territory. During this period, companies usually implement expansion-oriented strategies, which lead to relatively high demand for capital, while the high capital demand caused by the long continuous cycle of green innovation also requires a high-quality capital supply [34,49], a crowding effect may occur. In terms of the utility of green innovation, the expected results of green innovation are highly uncertain, and it is a risky investment for growth-stage enterprises. The short-term benefits that can be achieved by using funds for green innovation investment are lower than the income that can be brought from investing capital in production and sales [50]. It may go against the company’s strategic intention of increasing market share at the current stage, thus the subjective initiative of growing companies to carry out green innovation is relatively weak. For enterprises in the mature stage, their product production and sales tend to be stable. At this time, through green innovation, enterprises can not only improve the utilization efficiency and recycling rate of raw materials, and reduce resource costs and environmental costs [51,52], but can also draw consumers’ attentions to the corporate environmental behavior, establish a good corporate image, and promote the growth of enterprise developable performance [53]. Compared with companies in the growth stage, companies in the mature stage are relatively complete in all aspects. With the help of the development of digital inclusive finance, they have more R&D strength and risk-taking ability to carry out green projects with high capital requirements and high-risk levels. Therefore, from the perspective of a life cycle, the high growth of enterprises may not be conducive to the promotion of digital inclusive finance for green innovation; that is, the development of digital inclusive finance may have a more significant incentive effect on the green innovation of enterprises in the mature stage of development.

To confirm this analysis, this paper refers to the practice of Liang Shangkun et al. [54] and ranks the growth rate of corporate sales revenue and capital expenditure rate from high to low and the retained rate of return and corporate age from low to high. The comprehensive score is calculated by assigning values, and then the comprehensive score is sorted and classified according to the industry. Companies belonging to the growth stage and mature stage are screened out, and the heterogeneous impact of digital inclusive finance on green innovation is tested in groups.

As shown in Table 6, the coefficient of digital financial inclusion in the growth stage group is 0.1802, and the coefficient of digital financial inclusion in the mature stage group is 0.4146, both of which are significant. This indicates that whether firms are in the growth stage or the mature stage, digital inclusive finance can have an incentive effect on green innovation. Furthermore, the grouping regression results obtained support the conclusion that the two are comparable through a seemingly uncorrelated test; that is, the predigital financial inclusion coefficient in the mature group is higher than the predigital financial inclusion coefficient in the growth period, which indicates that digital financial inclusion is important for green innovation. The promotion effect is stronger in mature enterprises.

## 5. Conclusions and Recommendations

### 5.1. Research Conclusions

Based on the green patent application data of listed companies in Shanghai and Shenzhen, A-share heavy polluting industries from 2011 to 2020 and the digital financial inclusion index compiled and released by the Financial Research Center of Peking University, this paper empirically analyzes the effect of digital financial inclusion on the green innovation of heavily polluting companies. The impact effect and its specific impact mechanism are further explored for life cycle heterogeneity. The main conclusions are as follows:

(1) Digital financial inclusion effectively stimulates the green innovation of heavily polluting enterprises, and the conclusion still holds after considering endogeneity and robustness.
(2) Digital financial inclusion alleviates corporate financing constraints, and the traditional problem of financial mismatch in financial services promotes the green innovation of heavily polluting enterprises.
(3) From the perspective of a life cycle, the effect of digital inclusive finance on the green innovation of heavily polluting enterprises in the mature stage is higher than that of those in the growth stage.

From the above conclusions, it can be seen that this study reveals the influencing mechanism of digital financial inclusion on the green innovation of heavy-polluting enterprises from the perspective of financing constraints and financial mismatch. It not only expands the relevant literature on the impact of digital financial inclusion on enterprises, but also provides a new perspective for analyzing the influencing factors of corporate green innovation. This study also clarifies the differences in the effects of digital financial inclusion on corporate green innovation at different development stages. However, this study also has certain limitations, mainly due to the availability of data. Moreover, the impact of the COVID-19 pandemic on green technology innovation in heavy-polluting enterprises was not considered in the research process. This is also an area worthy of further research in the future.

### 5.2. Policy Suggestions

This paper not only reveals the impact of the development of digital inclusive finance on the green innovation of heavily polluting enterprises but also clarifies its influence mechanism, which has important policy implications for promoting the green innovation of heavily polluting enterprises and the development of digital inclusive finance in China. First, the financing support system for green innovation of enterprises should be improved to promote the green transformation of heavily polluting enterprises. Green innovation in heavily polluting enterprises requires stable and continuous financial support. However, due to the nature of heavily polluting enterprises and the influence of national policies, obtaining financing is difficult because they face severe financing constraints and are easily affected by the misallocation of financial resources. Green innovation requires significant funds, thus the government should improve financing mechanisms to support the green innovation of heavily polluting enterprises. This will further encourage and support the development of digital inclusive finance through the application of information technology to support the financial needs for green innovation of heavily polluting enterprises. It will also improve the financing support mechanisms through green credit and tax incentives and the timely introduction of government subsidies to support the capital requirements for green innovation of heavily polluting enterprises. Second, for heavily polluting enterprises, it is necessary to strengthen their green business philosophy, enhance their environmental protection awareness, improve their information disclosure, reduce the degree of information asymmetry in the financing market, improve their financing capabilities, and avoid green credit. The punishment mechanism makes heavily polluting enterprises vulnerable to financing difficulties, resulting in insufficient funding sources for green innovation. Finally, for financial institutions such as banks, the channels for providing financial support to heavily polluting enterprises should be improved, including special loans to heavily polluting enterprises to support the adoption of green technology innovation.

## Figures and Tables

**Table 1:** Descriptive statistics of the main variables.

Variable	N	Mean	Std.Dev	Min	Max
Patent	6611	0.736	1.018	0	3.970
Dindex	6611	2.026	0.718	0.446	3.208
Size	6611	22.270	1.344	19.950	26.270
Lev	6611	0.426	0.209	0.051	0.950
Roa	6611	0.038	0.057	−0.193	0.204
PPe	6611	0.311	0.172	0.022	0.772
Dual	6611	1.756	0.429	1	2
Msh	6611	0.127	0.201	0	0.679
Top	6611	59.18	15.39	22.15	91.58
Balance	6611	0.874	0.766	0.035	3.772

**Table 2:** Correlation coefficient matrix.

Variable	VIF	Patent	Dindex	Size	Lev	Roa	PPe	Dual	Msh	Top	Balance
Patent	-	1									
Dindex	1.10	0.181 ***	1								
Size	1.81	0.487 ***	0.097 ***	1							
Lev	1.87	0.192 ***	−0.120 ***	0.486 ***	1						
Roa	1.35	0.00500	0.083 ***	−0.022 *	−0.432 ***	1					
PPe	1.30	0.124 ***	−0.112 ***	0.352 ***	0.398 ***	−0.209 ***	1				
Dual	1.10	0.117 ***	−0.108 ***	0.207 ***	0.162 ***	−0.054 ***	0.179 ***	1			
Msh	1.42	−0.125 ***	0.109 ***	−0.363 ***	−0.355 ***	0.198 ***	−0.309 ***	−0.243 ***	1		
Top	1.23	0.096 ***	0.067 ***	0.205 ***	−0.116 ***	0.247 ***	−0.041 ***	−0.045 ***	0.206 ***	1	
Balance	1.09	−0.061 ***	0.127 ***	−0.156 ***	−0.176 ***	0.080 ***	−0.150 ***	−0.050 ***	0.233 ***	0.00200	1

Note: *, **, and *** indicate significance at the 10%, 5%, and 1% levels, respectively.

**Table 3:** Digital financial inclusion and green innovation.

Variable	(1)	(2)	(3)	(4)
	Patent	Patent	2SLS Regression	
			Dindex	Patent
			First	Two
Dindex	0.2882 ***	0.0031 ***		0.3310 ***
	(5.23)	(6.18)		(6.06)
Tool			1.0255 ***	
			(248.05)	
Size		0.3991 ***	0.0006	0.4128 ***
		(35.05)	(0.93)	(32.34)
Lev		−0.2052 ***	−0.0056	−0.1671 **
		(−3.11)	(−1.29)	(−2.25)
Roa		−0.2130	−0.0237 *	−0.0979
		(−1.06)	(−1.69)	(−0.44)
PPe		−0.3551 ***	−0.0104 **	−0.3956 ***
		(−4.51)	(−2.15)	(−4.57)
Dual		0.1094 ***	−0.0038 **	0.1350 ***
		(4.63)	(−2.11)	(5.14)
Msh		0.0764	0.0059	0.1128 *
		(1.38)	(1.51)	(1.78)
Top		−0.0010	−0.0001 **	−0.0012
		(−1.37)	(2.28)	(−1.42)
Balance		−0.0234 *	−0.0003	−0.0267 *
		(−1.68)	(0.31)	(−1.70)
Cons	0.5690 ***	−8.5870 ***	0.0301	−9.6591 ***
	(8.13)	(−34.52)	(1.54)	(−30.23)
Year	YES	YES	YES	YES
Ind	YES	YES	YES	YES
N	6611	6611	5516	5516
Adj-R^2^	0.0976	0.3010	0.9932	0.2954

Note: Robust standard errors are in parentheses. *, **, and *** indicate significance at the 10%, 5%, and 1% levels, respectively.

**Table 4:** Robustness test.

Variable	(1)	(2)	(3)
	RD	Patent	Patent
Dindex	1.0832 ***		
	(10.77)		
PDindex		0.1780 ***	
		(4.29)	
L.Dindex			0.3310 ***
			(6.06)
Controls	YES	YES	YES
Cons	9.0161 ***	−8.5056 ***	−9.6591 ***
	(17.42)	(−34.33)	(−32.26)
Year	YES	YES	YES
Ind	YES	YES	YES
N	5399	6611	5516
Adj-R^2^	0.2984	0.2989	0.3029

Note: Robust standard errors are in parentheses. *, **, and *** indicate significance at the 10%, 5%, and 1% levels, respectively.

**Table 6:** Heterogeneity test.

Variable	(1)	(2)
	Growth	Maturity
	Patent	Patent
Dindex	0.1802 *	0.4146 ***
	(1.80)	(4.66)
Controls	YES	YES
Cons	−9.0829 ***	−8.3315 ***
	(−17.28)	(−20.49)
Year	YES	YES
Ind	YES	YES
N	1618	2106
Adj-R^2^	0.2910	0.3174
Suest	chi2(1) = 3.16 Prob > chi2 = 0.0754	

Note: Robust standard errors are in parentheses. *, **, and *** indicate significance at the 10%, 5%, and 1% levels, respectively.

## Data Availability

The data presented in this study are openly available in the China Research Data Service Platform and the Guotai Security Database.
